# Cytoskeletal Regulation of Podosome-Focal Adhesion Balance in GM-CSF- and Flt3L-Derived Dendritic Cells

**DOI:** 10.3390/cells15121125

**Published:** 2026-06-22

**Authors:** Zuzanna Biernacka, Karolina Gregorczyk-Zboroch, Iwona Lasocka, Michalina Bartak, Małgorzata Gieryńska, Justyna Struzik, Felix N. Toka, Lidia Szulc-Dąbrowska

**Affiliations:** 1 Department of Preclinical Sciences, Institute of Veterinary Medicine, Warsaw University of Life Sciences-SGGW, 02-786 Warsaw, Poland; zuzanna_biernacka@sggw.edu.pl (Z.B.); karolina_gregorczyk-zboroch@sggw.edu.pl (K.G.-Z.); michalina_bartak@sggw.edu.pl (M.B.); malgorzata_gierynska@sggw.edu.pl (M.G.); justyna_struzik@sggw.edu.pl (J.S.); ftoka@rossvet.edu.kn (F.N.T.); 2 Department of Biology of Animal Environment, Institute of Animal Science, Warsaw University of Life Sciences-SGGW, 02-786 Warsaw, Poland; iwona_lasocka@sggw.edu.pl; 3 Center for Integrative Mammalian Research, Ross University School of Veterinary Medicine, Basseterre P.O. Box 334, Saint Kitts and Nevis

**Keywords:** dendritic cells, adhesion structures, cytoskeletal dynamics

## Abstract

Dendritic cells (DCs) are key antigen-presenting cells essential for the initiation of immune responses. Their migration is tightly regulated by adhesive structures, including podosomes and focal adhesions (FAs), allowing for interactions with the extracellular matrix (ECM) for coordinated cell movement. The organization and dynamics of these structures are controlled by actin and microtubule cytoskeletons; however, the mechanisms governing their balance in distinct DC subsets are not completely understood. In this study, we investigated cytoskeletal regulation of the interplay between podosomes and FAs in GM-CSF-derived inflammatory-like DCs (GM-BMDCs) and Flt3L-derived conventional DCs (FL-BMDCs). GM-BMDCs showed a higher capacity to form podosomes compared with FL-BMDCs, which exhibited fewer and less prominent structures. Actin depolymerization resulted in the complete loss of podosomes, whereas disruption of microtubules induced podosome reorganization and altered the structure of FAs. Importantly, cytoskeletal perturbation in both DC subsets led to podosome dissolution, highlighting the requirement of cytoskeletal integrity for their maintenance. Furthermore, actin integrity was essential for podosome-mediated ECM degradation and efficient migration of GM-BMDCs, while microtubules fine-tuned the balance between podosome and focal adhesion dynamics, thereby regulating DC motility.

## 1. Introduction

Cell adhesion structures mechanically link the external microenvironment to the cellular cytoskeleton and play an important role in regulating many biological processes, such as cell signaling, tissue development, wound healing, and immune response during physiological and pathological conditions. These structures are formed between adjacent cells or a cell and an extracellular matrix (ECM). Various types of cell–matrix adhesion structures have been classified within different cells based on their organization, dynamics, location, protein composition, and proteolytic capability, ranging from classical focal adhesions (FAs) to podosomes and podosome-type structures, called invadopodia, formed by invasive cancer cells [1].

Although FAs and podosomes share most of the same proteins, their architecture, dynamics, and functions are fundamentally different. FAs are elongated structures physically coupled with actin stress fibers that mediate stable cell adhesion to the ECM [2,3,4]. FAs are considered primary sites for cellular force transduction and regulatory signal transmission that control cell behavior and drive cell migration [5]. In contrast, podosomes are circular structures composed of a protrusive actin-rich core surrounded by an adhesive integrin-rich ring [6]. Single podosomes can organize in more complex structures or superstructures called clusters, rosettes, or belts in different cell types [7,8]. Podosomes are more unstable and dynamic than FAs and are formed by highly motile cells. Apart from their close involvement in cell motility, adhesion, and mechanosensing (similar to FAs), podosomes are also responsible for extracellular matrix degradation and antigen sampling. The latter phenomenon was observed in highly specialized immune system cells, called dendritic cells (DCs) [9].

DCs link the innate and adaptive immunity by influencing the type, magnitude, and quality of the specific immune response during infectious and non-infectious diseases. In their immature state, DCs reside in lymphoid and peripheral tissues where they survey for foreign antigens. In this state, DCs are highly endocytic and exhibit a slow mesenchymal mode of migration, allowing them to explore the microenvironment. They can easily migrate through tissues and cross tissue barriers, as they can form podosomes and FAs at this stage. After antigen recognition and uptake, DCs become activated, lose their endocytic activity, and start to perform a fast ameboid mode of migration, which is associated with podosome dissolution [10]. The ability of DCs to dissolve podosomes and transition to amoeboid movement is crucial for their rapid migration to the draining lymph nodes, where they function as antigen-presenting cells (APCs) and activate antigen-specific T cells.

Because DCs are highly migratory cells and travel within the body through “crawling” between cells, the activity of adhesion structures is tightly regulated by interaction between the actin cytoskeleton and microtubules (MTs). It has been shown that depolymerization of MTs using nocodazole, a synthetic MT-disrupting drug, resulted in an increase in the number and the size of FAs in different immune and non-immune cells, including macrophages [11] and fibroblasts [12,13], respectively. Such a phenomenon was dependent on the activation of the small G protein Rho, because the downregulation of Rho plays a role in the disassembly of FAs [12,14]. In contrast to FAs, the effect of nocodazole treatment on podosome formation was more cell-specific and resulted in the rapid and complete disassembly of podosomes in primary human monocytes and macrophages [11] or led to the disorganization of podosome structures in osteoclasts [15]. In the latter case, microtubule depolymerization using nocodazole was associated with a disruption of the podosome belt, which is normally observed as a narrow circular band of podosomes at the cell periphery in mature multinucleated osteoclasts [15]. The podosome belt was replaced by several podosome rings and clusters, which seemed to be resistant to microtubule depolymerization [15]. Rho inhibition results in the maintenance of the podosome belt despite MT destruction with nocodazole, suggesting that the relationship between Rho inhibition and podosomes is certainly different from that with FAs [16].

Currently, there is limited information regarding the role of the cytoskeleton in regulating the balance between focal adhesions (FAs) and podosome turnover in dendritic cells (DCs). In the present study, we used the most commonly applied in vitro dendritic cell (DC) models. These included inflammatory-like dendritic cells generated in a granulocyte-macrophage colony-stimulating factor (GM-CSF)-dependent system. These cells resemble monocyte-derived, inflammation-associated DC populations. We also used conventional dendritic cells derived in an Fms-like tyrosine kinase 3 ligand (Flt3L)-dependent system. These cells recapitulate steady-state classical DC subsets involved in immune surveillance.

We demonstrate a microtubule-dependent turnover of adhesion structures in primary murine inflammatory-like and conventional DC populations. Similar to other cells, microtubule depolymerization resulted in increased size and stability of FAs in primary DC cultures. We further show that intact microtubules are necessary for the generation of highly organized podosome clusters in DCs and for the proper migration of these cells in a two-dimensional microenvironment.

## 2. Materials and Methods

### 2.1. Animals

Male Balb/c (H-2d) mice were purchased from the animal facility at the Maria Skłodowska-Curie Institute-Oncology Centre in Warsaw. Bone marrow was collected from animals aged between 8 to 12 weeks after cervical dislocation; therefore, approval from the Local Ethical Committee was not required. All experiments were conducted in accordance with the institutional guidelines for the care and use of laboratory animals.

### 2.2. Reagents

Mouse recombinant granulocyte-macrophage colony-stimulating factor (rmGM-CSF; Sigma-Aldrich, St. Louis, MO, USA) and mouse recombinant FMS-like tyrosine kinase 3 ligand (rmFlt3L; Sigma-Aldrich) were used to obtain a primary culture of bone marrow-derived dendritic cells (BMDCs). For cytoskeletal perturbation experiments, cytochalasin D, nocodazole, and taxol (all from Sigma-Aldrich) were dissolved in dimethyl sulfoxide (DMSO). Positive magnetic cell separation was performed using MACS UltraPure MicroBeads conjugated to monoclonal anti-mouse CD11c antibodies (Miltenyi Biotec, Auburn, CA, USA). For immunofluorescence staining, F-actin was visualized using phalloidin conjugated with FITC or Rhodamine Red-X (Sigma-Aldrich). Primary antibodies against vinculin and α-tubulin (Sigma-Aldrich) were used together with species-specific secondary antibodies conjugated to FITC or Rhodamine Red-X (Jackson ImmunoResearch Laboratories, West Grove, PA, USA). Nuclear staining was performed using Hoechst 33342 (Sigma-Aldrich), and samples were mounted using ProLong Gold Antifade Mountant (Thermo Fisher Scientific, Waltham, MA, USA). For extracellular matrix degradation assays, FITC-conjugated fibronectin (Sigma-Aldrich) was used in combination with poly-L-lysine and glutaraldehyde (Sigma-Aldrich) for coverslip coating.

### 2.3. Isolation of Bone Marrow (BM) Cells

Mouse recombinant granulocyte-macrophage colony-stimulating factor (rmGM-CSF; Sigma-Aldrich, St. Louis, MO, USA) and mouse recombinant FMS-like tyrosine kinase 3 ligand (rmFlt3L; Sigma-Aldrich) were used to obtain a primary culture of bone marrow-derived dendritic cells (BMDCs), as described previously [8]. Briefly, tibias and femurs were isolated from Balb/c mice, and bone marrow cells were flushed using RPMI 1640 medium (Gibco, Grand Island, NY, USA) supplemented with 1% antibiotic solution (100 U/mL penicillin and 100 µg/mL streptomycin, Symbios, Gdansk, Poland). A single-cell suspension was prepared by dispersing bone marrow cell aggregates. After erythrocyte lysis with NH_4_Cl-Tris buffer, the cells were washed and resuspended in an appropriate growth medium.

### 2.4. Generation of BMDCs Using GM-CSF

R-10 growth medium [RPMI-1640 medium enriched with 10% heat-inactivated fetal bovine serum (FBS, HyClone, Logan, UT, USA), 1% antibiotic solution, and 50 μM 2-mercaptoethanol (Sigma-Aldrich)] supplemented with 20 ng/mL rmGM-CSF was used for the generation of inflammatory-like BMDCs (referred to as GM–BMDCs). The cells were seeded into wells of a six-well plate and incubated for 8 days at 37 °C in a humidified 5% CO_2_ atmosphere. R-10 medium was added on day 3, followed by a partial medium replacement on day 6. After cultivation, DCs were enriched using positive magnetic cell separation with MACS UltraPure MicroBeads conjugated to monoclonal anti-mouse CD11c antibodies (Miltenyi Biotec). Cell viability was greater than 95%, as determined by the 0.4% trypan blue exclusion assay. The population purity of separated cells was confirmed by flow cytometry for surface marker expression, as previously described [17].

### 2.5. Generation of BMDCs Using Flt3L

R-10 growth medium supplemented with 200 ng/mL rmFlt3Lwas used for the generation of BMDCs (referred to as FL–BMDCs). The cells were seeded into wells of a six-well plate, incubated for 9 days (37 °C, 5% CO_2_), and used in further experiments. Cell viability was greater than 95%, as determined by the 0.4% trypan blue exclusion assay. Cells were confirmed for the expression of CD11c, CD11b, MHC II, CD80, CD86 surface markers by flow cytometry.

### 2.6. Treatment of BMDCs with Cytoskeleton-Interfering Agents

GM-BMDCs and FL-BMDCs were seeded at 2 × 10^5^ on a glass coverslip in a 24-well culture plate and were treated or not with 1% DMSO, 0.5 μM cytochalasin D, 20 μM nocodazole, and/or 5 µM taxol dissolved in DMSO. The cells were incubated for 2 h, 24 h, or for 2 h followed by washing with R-10 medium, and subsequently cultured for 24 h at 37 °C in a humidified 5% CO_2_ atmosphere. Then, the cells were fixed in cold methanol or 2% PFA (Sigma-Aldrich) and subsequently labeled for fluorescence microscopy.

### 2.7. Immunofluorescence Staining

To characterize podosomes and FAs in GM-BMDCs and FL-BMDCs, F-actin was labeled with phalloidin conjugated with FITC or Rhodamine Red-X (both from Sigma-Aldrich). Vinculin and α-tubulin were visualized using antigen (Ag)-specific unlabeled mouse primary antibodies (Sigma-Aldrich) and secondary anti-mouse IgG antibodies conjugated to Rhodamine Red-X or FITC (Jackson ImmunoResearch Laboratories, West Grove, PA, USA). The cellular DNA was stained using Hoechst 33342 dye (Sigma-Aldrich). Microscopic slides were embedded in ProLong Gold Antifade Mountant (Thermo Fisher Scientific, Waltham, MA, USA).

### 2.8. ECM Degradation Assay

Glass coverslips were coated with 50 μg/mL poly-L-lysine (Sigma-Aldrich, St. Louis, MO, USA), 0.5% glutaraldehyde, and a gelatin/fibronectin-FITC mixture (Sigma-Aldrich). Each step was followed by three washes with sterile PBS. Before cell seeding, the slides were disinfected with 70% ethanol and equilibrated with R-10 medium. GM-BMDCs were placed at 2 × 10^5^ cells/well on glass coverslips coated with FITC-conjugated fibronectin and were treated or not with 1% DMSO, 0.5 μM cytochalasin D, 20 μM nocodazole, and 5 µM taxol dissolved in DMSO. The cells were incubated for 24 h or for 2 h followed by washing with R-10 medium and subsequently cultured for 24 h at 37 °C in a humidified 5% CO_2_ atmosphere. The cells were fixed in 2% PFA for further analysis.

### 2.9. Fluorescence Microscopy Analysis

Fluorescence microscopy analysis was performed on at least 50 or 100 randomly selected single cells per slide for each experimental condition. Only isolated cells without direct cell-to-cell contact were included to avoid potential interference with the accurate quantification of FAs per cell. For each experimental condition, the length of at least 250 FAs was measured using ImageJ 1.52a (NIH, Bethesda, MD, USA). All FAs from each randomly selected cell were included in the measurement. Adhesive structures were analyzed using an Olympus BX60 fluorescence microscope (Olympus, Tokyo, Japan) equipped with a PROMICAM 3-5CP camera and QuickPHOTO 2.3 software (Promicra, Prague, Czech Republic). Brightness and contrast were adjusted using CellSens Dimension 1.9 software (Olympus) or Adobe Photoshop CS2 (Adobe Systems Inc., San Jose, CA, USA).

### 2.10. Wound-Healing Assay

The motility of GM-BMDCs was assessed using a wound-healing assay. Cells were seeded onto a glass coverslip in a 24-well culture plate to form a monolayer (5 × 10^5^ cells/well). A scratch running through the center of the slide was made on the cell monolayer using a sterile micropipette tip. Subsequently, the medium was replaced to wash the floating cells off. Cells were treated or not with 1% DMSO, 0.5 μM cytochalasin D, 20 μM nocodazole, and 5 µM taxol dissolved in DMSO. Wound overgrowth by GM-BMDCs was observed at 0 and 24 h of culture in R-10 medium at 37 °C in a humidified 5% CO_2_ atmosphere using an Olympus IX71 inverted microscope (Olympus) equipped with a PROMICAM 3-5CP camera and QuickPHOTO software. Cell migration was quantified as the difference between the number of cells within the wound at 24 h and 0 h. The cells were incubated for 24 h (37 °C, 5% CO_2_) and subsequently fixed in 2% PFA (Sigma-Aldrich, St. Louis, MO, USA) for further analysis.

### 2.11. Statistical Analysis

The results are presented as arithmetic means ± standard deviation (SD) from at least three independent experiments. Statistical analysis was performed using paired or unpaired Student’s *t*-test using Statistica 13.2 software (StatSoft Inc., Tulsa, OK, USA). Data were tested for normality using the Kolmogorov–Smirnov test. Statistical significance was set at α = 0.05, and determined as *p* < 0.05 (*), *p* < 0.01 (**), and *p* < 0.001 (***).

## 3. Results

### 3.1. Podosome Organization and FA Characteristics in GM-CSF- and Flt3L-Derived BMDCs

**Figure 1:**
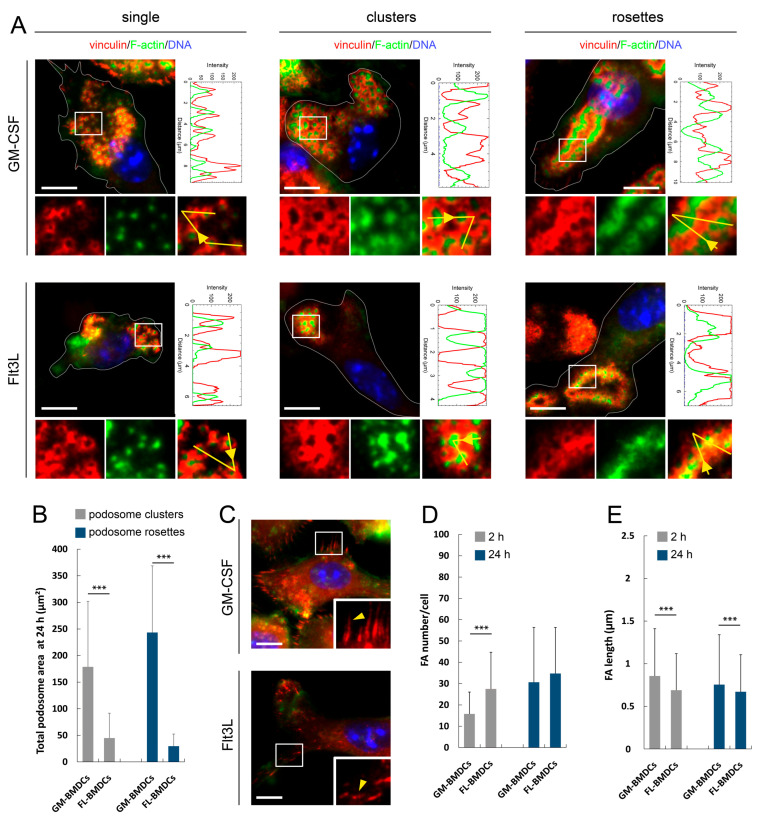
Formation and organization of adhesive structures in BMDCs differentiated with GM-CSF (GM-BMDCs) and Flt3L (FL-BMDCs) under control conditions. (**A**) Representative images from fluorescence microscopy showing three podosome organization patterns: single podosomes, podosome clusters, and podosome rosettes. Cells were stained for vinculin (red), F-actin (green), and DNA (blue). White outlines indicate cell boundaries. Magnified images show the regions indicated by boxes. Intensity profile plots show the spatial distribution of vinculin and F-actin along the indicated line scans. Yellow arrowheads indicate the direction along which the fluorescence intensity profile was measured. Original magnification: 1500×. Scale bars: 5 µm. (**B**) Quantification of the total area occupied by podosome clusters and podosome rosettes per cell in cells after 24 h of culture. (**C**) Representative high-magnification images of focal adhesions (FAs). Insets show enlarged regions containing individual FAs (yellow arrowheads). Original magnification: 1500×. Scale bars: 5 µm. (**D**) Quantification of the number of focal adhesions (FAs) per cell and (**E**) FA length after 2 h and 24 h of culture. Podosome size, FA number per cell, and FA length were quantified from at least 50 cells and 1000 FAs randomly selected for each experimental condition across three independent experiments. Data represent mean ± standard deviation (SD) from three independent experiments. Comparisons between groups were performed using an unpaired Student’s *t*-test. Horizontal lines indicate the compared groups; asterisks above the lines denote statistical significance (*** *p* < 0.001).

To evaluate differences in adhesive structures between GM-BMDCs and FL-BMDCs the organization of podosomes and FAs was analyzed by fluorescence microscopy (Figure 1). In both types of cells, podosomes were characterized by a typical structure, i.e., a podosome core composed of F-actin and a surrounding ring composed of the adaptor protein vinculin (Figure 1A). Based on their morphological organization and complexity, podosomes were classified into single, clustered, and rosette forms, the latter of which represent the most complex and mature structures. Single podosomes were characterized by a loosely connected structure, either evenly distributed over the entire basal surface of the cell or localized to specific regions of the cell bottom. Single podosomes were separated from each other by empty spaces in which no adhesion structures were observed. Podosome clusters were classified as dense, well-organized podosome structures without empty spaces between individual podosomes. Podosome clusters were observed mainly on the basal surface of the cell. Rosettes were classified as superstructures characterized by large ring-like bands composed of vinculin, surrounding on both sides a central band consisting of densely packed actin puncta (Figure 1A). Quantitative analysis demonstrated that GM-BMDCs exhibit a significantly larger area occupied by podosome clusters and podosome rosettes compared to FL-BMDCs (Figure 1B).

The formation of FAs was further assessed at 2 h and 24 h after cell seeding (Figure 1C). In both types of cells, FAs were elongated, well-organized, and colocalized with actin fibers (Figure 1C). The number of FAs per cell varied over the culture period, averaging 16 after 2 h and 31 after 24 h in GM-BMDCs, and 28 after 2 h and 34 after 24 h in FL_BMDCs (Figure 1D). FL-BMDCs displayed a significantly higher number of FAs per cell at 2 h compared with GM-BMDCs; however, this difference was no longer observed after 24 h of culture (Figure 1D). In contrast, FA length was consistently greater in GM-BMDCs than in FL-BMDCs at both analyzed time points (Figure 1E). These results suggest that GM-BMDCs preferentially form larger and more elongated adhesive structures, whereas FL-BMDCs generate a greater number of smaller FAs during the early stages of surface attachment.

### 3.2. Cytoskeletal Inhibitors Alter Microtubule Architecture in GM-BMDCs and FL-BMDCs

**Figure 2:**
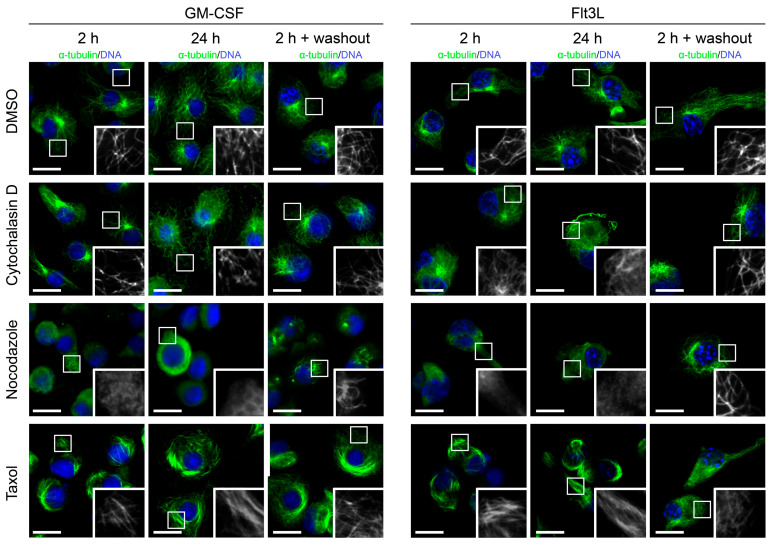
Microtubule organization in GM-BMDCs and FL-BMDCs following treatment with actin and tubulin cytoskeleton inhibitors. Cells treated with DMSO (control), cytochalasin D, nocodazole, or taxol were stained for α-tubulin (green) and DNA (blue) after 2 h and 24 h of treatment, as well as after 2 h treatment followed by washout. Magnified images show the regions indicated by boxes. Original magnification: 1500×. Scale bars: 12 µm.

The effects of cytoskeletal inhibitors (cytochalasin D, nocodazole, and taxol) on the morphology of the tubulin cytoskeletal network were evaluated in GM-BMDCs and FL-BMDCs (Figure 2). Cytochalasin D was used as a potent inhibitor of actin polymerization by blocking barbed-end elongation of actin filaments, thereby disrupting actin-based structures. Nocodazole was applied as a microtubule-depolymerizing agent that disrupts microtubule dynamics by inhibiting tubulin polymerization, whereas taxol (paclitaxel) was used as a microtubule-stabilizing compound that suppresses microtubule dynamics and interferes with microtubule turnover.

In control (DMSO-treated) GM-BMDCs and FL-BMDCs, microtubules formed a dynamic, radially organized network emanating from the perinuclear microtubule-organizing center (MTOC) toward the cell periphery, which became progressively more elaborated with culture time, particularly after 24 h. In both cell types, cytochalasin D, a potent inhibitor of actin polymerization, induced marked reorganization of the microtubule system, leading to the formation of a disorganized, entangled network with localized microtubule thickening after 24 h of treatment, with partial reversibility observed upon washout. Nocodazole treatment resulted in rapid microtubule depolymerization and fragmentation, leading to loss of radial architecture and diffuse cytoplasmic tubulin distribution, with partial reassembly and restoration of MTOC-centered organization following drug removal. In contrast, taxol stabilized microtubules, promoting the formation of highly ordered, thickened, and concentrically arranged bundles surrounding the nucleus, evident as early as 2 h and persisting up to 24 h, with gradual recovery of the canonical MTOC-dependent radial organization after washout. These data confirm that the organization of the tubulin cytoskeleton in both types of BMDCs is highly dynamic and tightly regulated by the interplay between actin integrity and microtubule polymerization stability.

### 3.3. Cytoskeletal Regulation of Podosome Formation in GM-BMDCs Under Modulation of Actin and Microtubule Dynamics

**Figure 3:**
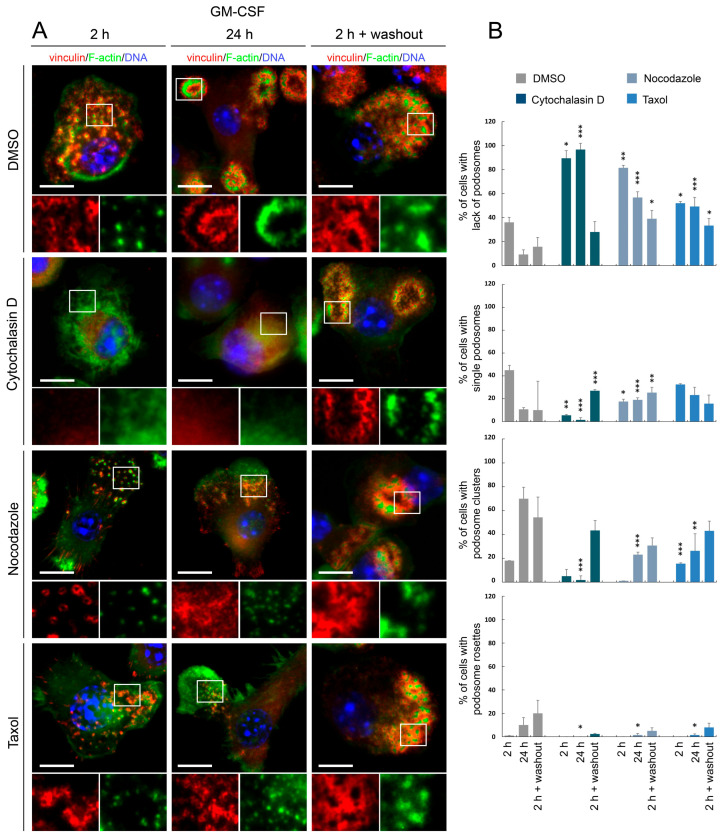
Cytoskeletal-inhibitor-induced disassembly and disintegration of podosomes in GM-BMDCs. (**A**) Fluorescence microscopy analysis of podosomes in GM-BMDCs. Cells treated with DMSO (control), cytochalasin D, nocodazole, or taxol were stained for vinculin (red), F-actin (green), and DNA (blue) after 2 h and 24 h of treatment, as well as after 2 h of treatment followed by washout. Magnified images show the regions indicated by boxes. Original magnification: 1500×. Scale bars: 8 µm. (**B**) Mean percentage of cells lacking podosomes or containing single podosomes, clusters, or rosettes in cells treated with DMSO (control), cytochalasin D, nocodazole, or taxol after 2 h and 24 h of treatment, as well as after 2 h treatment followed by washout. Cell percentages were determined by counting at least 100 randomly selected cells per experimental condition. Data represent mean ± standard deviation (SD) from three independent experiments. Statistical significance was determined using an unpaired Student’s *t*-test. Asterisks above the columns indicate significant differences compared with the DMSO control at the corresponding time point: * *p* < 0.05, ** *p* < 0.01, *** *p* < 0.001.

In control GM-BMDCs, podosomes were present at all time points and displayed increasing structural complexity over time, transitioning from predominantly single, loosely distributed structures (~45% at 2 h) to mainly clustered forms (~70% at 24 h), with occasional rosettes (~10%). The overall percentage of podosome-containing cells increased from <40% at 2 h to ~90% at 24 h and remained stable after washout (Figure 3B).

Disruption of the cytoskeleton significantly impaired podosome formation and organization. Cytochalasin D induced rapid disassembly of podosomes, leading to their near-complete loss, with 90% of cells lacking podosomes at 2 h post treatment (hpt) (*p* < 0.05) and >96% at 24 hpt (*p* < 0.001), and only minimal presence of single podosomes (5.5%) without rosette formation. Microtubule-targeting agents (nocodazole and taxol) also reduced podosome presence and altered their organization, resulting in 82% (nocodazole, *p* < 0.01) and 52% (taxol, *p* < 0.05) of cells lacking podosomes after 2 h, with remaining cells exhibiting mainly single structures (17% and 32%, respectively) and no rosettes. After 24 h, podosome-positive cells remained significantly reduced (DMSO—90% vs. nocodazole—44% vs. taxol—50%; *p* < 0.001), and rosettes were nearly absent (~1%, *p* < 0.05). Morphologically, microtubule disruption caused cell rounding, loss of cytoplasmic actin organization, and predominance of poorly organized structures; however, some podosome formation persisted, confirming a regulatory rather than structural role of microtubules. Consistently, after nocodazole treatment, 18% of cells exhibited single podosomes at 2 h (*p* < 0.01), increasing at 24 h to 19% (single, *p* < 0.001) and 23% (clusters, *p* < 0.001), while taxol-treated cells showed a shift from single podosomes (32% at 2 h) to more clusters (26% at 24 h, *p* < 0.01), indicating partial recovery of complexity over time. Importantly, all effects were largely reversible, as washout restored actin organization and podosome formation in >80% of cells after cytochalasin D and 70% after nocodazole and taxol, predominantly as clusters at the leading edge, accompanied by reappearance of rosette structures (Figure 3A,B). These data suggest that podosome formation and maturation in GM-BMDCs are highly dependent on actin integrity and are modulated by microtubule dynamics, which play a regulatory role in their organization, stability, and spatial distribution.

### 3.4. Cytoskeletal Inhibitors Disrupt Podosome Organization and Dynamics in FL-BMDCs

**Figure 4:**
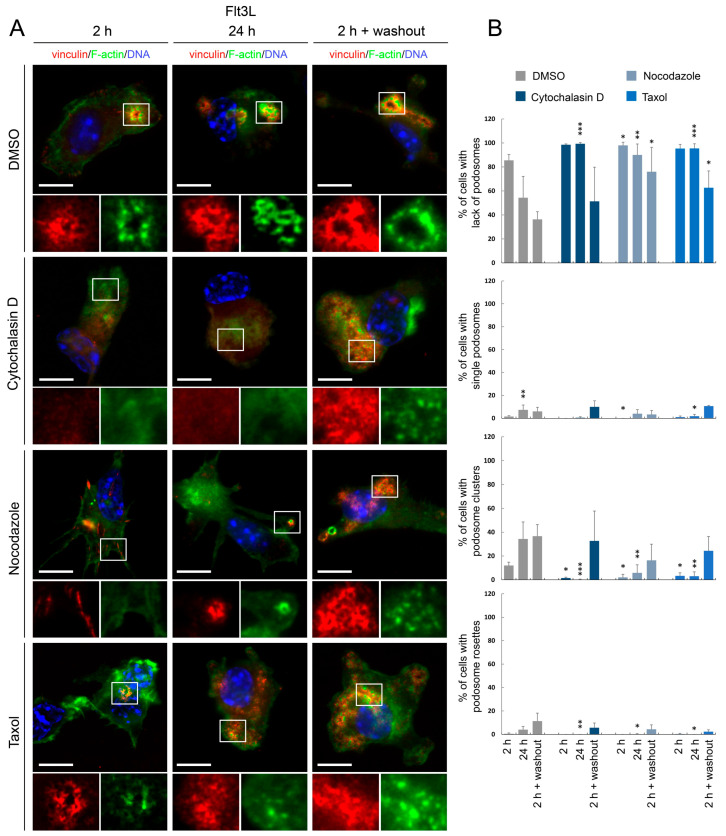
Cytoskeletal-inhibitor-induced disassembly and disintegration of podosomes in FL-BMDCs. (**A**) Fluorescence microscopy analysis of podosomes in dendritic cells. Cells treated with DMSO (control), cytochalasin D, nocodazole, or taxol were stained for vinculin (red), F-actin (green), and DNA (blue) after 2 h and 24 h of treatment, as well as after 2 h of treatment followed by washout. Magnified images show the regions indicated by boxes. Original magnification: 1500×. Scale bars: 8 µm. (**B**) Mean percentage of cells lacking podosomes or containing single podosomes, clusters, or rosettes in cells treated with DMSO (control), cytochalasin D, nocodazole, or taxol after 2 h and 24 h of treatment, as well as after 2 h treatment followed by washout. Cell percentages were determined by counting at least 100 randomly selected cells per experimental condition. Data represent mean ± standard deviation (SD) from three independent experiments. Statistical significance was determined using an unpaired Student’s *t*-test. Asterisks above the columns indicate significant differences compared with the DMSO control at the corresponding time point: * *p* < 0.05, ** *p* < 0.01, *** *p* < 0.001.

Subsequently, the formation and organization of podosomes were examined in DC cultures generated with Flt3L, which promotes the differentiation of different DC subsets, including conventional DCs, that closely resemble their physiological counterparts in vivo. Control cells treated with DMSO exhibited a regular, often elongated fibroblastic morphology with protrusions and well-defined cell polarization, and podosomes were observed at all time points (Figure 4A).

Similar to GM-BMDCs, podosomes formed primarily at the leading edge of cells, and their proportion increased over the course of culture. However, at each analyzed time point, the percentage of cells lacking podosomes was noticeably higher than in GM-BMDC cultures. Based on morphological organization, podosomes in FL-BMDCs were classified as single, clustered, and rosette forms (Figure 4A), similar to those in GM-BMDCs.

During the experiment, a gradual reorganization of podosomes was observed, characterized by a well-organized network structure, particularly at later culture stages (Figure 4A). The proportion of cells with podosome clusters increased (2 hpt—12%, 24 hpt—34%, 2 hpt + washout—23%), as did the proportion of cells with podosome rosettes (2 hpt—0.5%, 24 hpt—4%, 2 hpt + washout—11%) (Figure 4B). Simultaneously, the proportion of cells with single podosomes was also elevated (2 hpt—1.5%, 24 hpt—7%) (Figure 4B). Short-term exposure to DMSO followed by washout reduced the percentage of cells with single podosomes to approximately 6% (Figure 4B).

Treatment with cytochalasin D, nocodazole, or taxol strongly disrupted podosome organization and presence in FL-BMDCs. After 2 h, podosomes were nearly absent: 98% of cells lacked them after cytochalasin D and nocodazole, and 95% after taxol (*p* < 0.05) treatment. Cytochalasin D caused actin disorganization and cell rounding. Nocodazole induced irregular, “spiky” morphology and later excessive protrusion formation, while taxol caused rounding and cortical F-actin accumulation (Figure 4A).

After 24 h of cytochalasin D and taxol treatment, 95% of cells still lacked podosomes (*p* < 0.001). In nocodazole treated cells, this decreased to 90% (*p* < 0.01), with small proportions showing clusters (6%) and single podosomes (4%). Rosettes were absent under all inhibitor conditions. Residual podosomes had weak actin networks and were of smaller size.

Following 2 h of inhibitor treatment and 24 h recovery, podosome reformation occurred: 48% (cytochalasin D), 24% (nocodazole), and 38% (taxol). After washout, podosomes were present in 32%, 16%, and 24% of cells, respectively, without significant differences between the groups. Clusters predominated, and rosettes reappeared under all conditions.

### 3.5. Cytoskeletal Disruption in GM-BMDCs Modulates FA Number and Morphology in a Time-Dependent Manner

**Figure 5:**
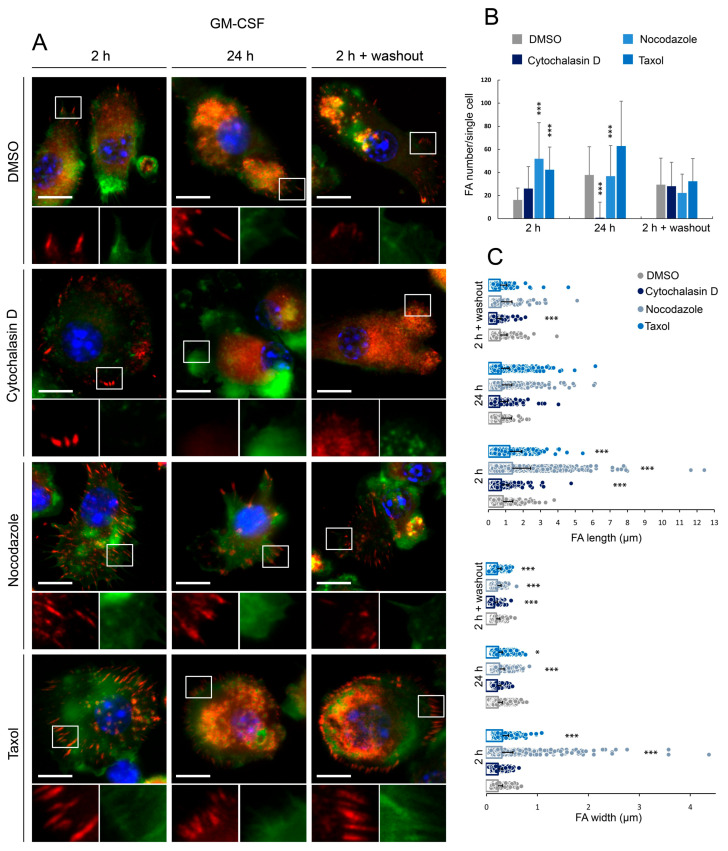
Cytoskeletal inhibitor–induced changes in cytoskeleton organization lead to alterations in the number, elongation, and widening of focal adhesions (FAs) in GM-BMDCs. (**A**) Fluorescence microscopy analysis of FAs in cells treated with DMSO (control), cytochalasin D, nocodazole, or taxol. Cells were stained for vinculin (red), F-actin (green), and DNA (blue) after 2 h and 24 h of treatment, and after 2 h followed by washout. Magnified images show the regions indicated by boxes. Original magnification: 1500×. Scale bars: 8 µm. (**B**) Number of FAs per cell and (**C**) FA length and FA width (both in µm) in cells treated with DMSO (control) and inhibitors after 2 h and 24 h of treatment, as well as after 2 h of treatment followed by washout. FA number per cell and their dimensions (length and width) were quantified from at least 50 cells and 1000 FAs, respectively, randomly selected for each experimental condition across three independent experiments. Each spot represents an individual data point, and bars indicate the mean values from three independent experiments. Statistical significance was determined using an unpaired Student’s *t*-test. Asterisks above the columns indicate significant differences compared with the DMSO control at the corresponding time point: * *p* < 0.05, *** *p* < 0.001.

Next, we assessed the dynamics of FA formation and their morphometric parameters in control GM-BMDCs and in cells treated with selected inhibitors of the actin and tubulin cytoskeleton. Short-term (2 h) cytochalasin D treatment led to shorter, more punctate focal adhesions (FAs) and increased their number (avg. 26 per cell), whereas long-term exposure (24 h) nearly abolished FAs (<1 per cell; *p* < 0.001) and disrupted actin stress fibers; partial reassembly occurred after washout, although FAs remained smaller. In contrast, short-term nocodazole treatment increased both FA size and number (avg. 54; *p* < 0.001), with more dispersed and stabilized structures, while after 24 h the FA number decreased (avg. 37; *p* < 0.001). Taxol also reduced FA dynamics, causing peripheral FA localization after 2 h and increasing both their number (avg. 41; *p* < 0.001) and size. After 24 h, FAs became smaller but more numerous (avg. 65) and evenly distributed at the cell periphery. Following short-term treatment and washout with an additional 24 h incubation, FA numbers returned to control levels (DMSO—29, cytochalasin D—28, nocodazole—22, taxol—32), although nocodazole- and taxol-treated cells showed reduced FA size and number compared to earlier time points, indicating partial regeneration of both microtubules and actin stress fibers (Figure 5A,B).

Morphometric analysis of FAs revealed that FA width remained stable at 0.24, 0.24, and 0.20 µm in control GM-BMDCs treated with DMSO, while FA length slightly decreased from 0.85 to 0.75 and 0.67 µm over time (Figure 5C). Microtubule inhibitors had the strongest effect: after 2 h, nocodazole and taxol significantly increased FA size (*p* < 0.001) to 1.38 × 0.31 µm and 1.23 × 0.32 µm, respectively, with nocodazole inducing extremely large FAs. In contrast, 2 h cytochalasin D treatment reduced FA length (0.75 vs. 0.85 µm; *p* < 0.001) without affecting width. After 24 h, FA length and width decreased to 0.77, 0.73, and 0.65 µm and 0.27, 0.24, and 0.22 µm for nocodazole, taxol, and cytochalasin D, respectively. Following washout, FA length remained unchanged for nocodazole and taxol, but width increased slightly (0.20 vs. 0.22 µm; *p* < 0.001), whereas cytochalasin D further reduced both length (0.73 vs. 0.48 µm) and width (0.20 vs. 0.16 µm; *p* < 0.001). Overall, the largest FAs were observed after short-term microtubule inhibition, highlighting the key role of microtubules in regulating FA growth and turnover.

### 3.6. Cytoskeletal Disruption in FL-BMDCs Modulates FA Number and Morphology in a Time-Dependent Manner

**Figure 6:**
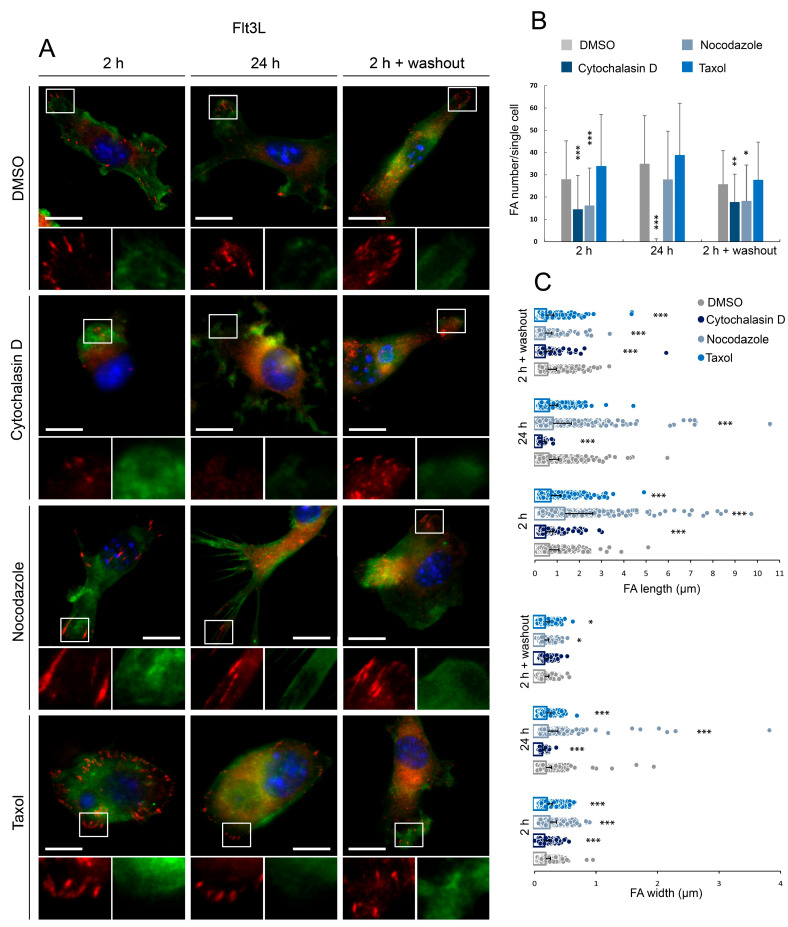
Cytoskeletal inhibitor–induced changes in cytoskeleton organization lead to alterations in the number, elongation, and widening of focal adhesions (FAs) in Flt3L-BMDCs. (**A**) Fluorescence microscopy analysis of FAs in control cells (DMSO) and cells treated with cytochalasin D, nocodazole, or taxol. Cells were stained for vinculin (red), F-actin (green), and DNA (blue) after 2 h and 24 h of treatment, and after 2 h followed by washout. Magnified images show the regions indicated by boxes. Original magnification: 1500×. Scale bars: 8 µm. (**B**) Number of FAs per cell and (**C**) FA length and FA width (both in µm) in control and inhibitor-treated cells after 2 h and 24 h of treatment, and after 2 h followed by washout. The number of FAs per cell was quantified in at least 50 randomly selected cells per experimental condition from three independent experiments. FA length and width were measured in at least 1000 FAs from randomly selected cells per experimental condition from three independent experiments. Each spot represents an individual data point, and bars indicate the mean values. Statistical significance was determined using an unpaired Student’s *t*-test. Asterisks above the columns indicate significant differences compared with the DMSO control at the corresponding time point: * *p* < 0.05, ** *p* < 0.01, *** *p* < 0.001.

A similar trend to that observed in GM-BMDCs was found in FL-BMDCs, with clear differences in FA number, organization, and morphometry across conditions (Figure 6A–C). DMSO-treated FL-BMDCs were polarized, with small FAs at the leading edge and larger, mature adhesions in the uropodium. Over time, cells became more flattened, with increased actin stress fibers and more mature FAs (Figure 6A). The mean FA number per cell reached 28, 34, and 25 at 2 hpt, 24 hpt, and after washout, respectively (Figure 6B). Morphometric analysis showed only minor changes, with FA length and width measuring 0.68 µm × 0.21 µm (2 hpt), 0.66 µm × 0.21 µm (24 hpt), and 0.61 µm × 0.19 µm (2 hpt + 24 h) (Figure 6C).

Cytoskeletal inhibitors significantly altered FA organization and size. Cytochalasin D disrupted actin structure, reduced FA number (14 at 2 h and 0 at 24 h; *p* < 0.001), and decreased FA size (0.51 µm × 0.19 µm at 2 h; 0.49 µm × 0.15 µm at 24 h; *p* < 0.001) (Figure 6A–C). In contrast, microtubule inhibitors, particularly nocodazole, had the strongest impact on FA morphometry, increasing FA length and width after 2 h (1.38 µm × 0.27 µm for nocodazole; 0.75 µm × 0.23 µm for taxol; *p* < 0.001) and maintaining elevated values at 24 h (0.85 µm × 0.24 µm and 0.68 µm × 0.22 µm, respectively), with nocodazole promoting larger, more stable FAs despite their lower number (Figure 6A–C). Taxol induced rounded, non-polarized cells with evenly distributed FAs and a slight, non-significant increase in FA number (33 at 2 h; 27 at 24 h) (Figure 6A,B). After washout, FA numbers normalized in taxol-treated cells, whereas cytochalasin D and nocodazole resulted in fewer, less mature adhesions (17 and 18 vs. 25 in control; *p* < 0.01 and *p* < 0.05) (Figure 6A,B), and FA length decreased significantly in all conditions (*p* < 0.001). FA width remained comparable between DMSO (0.195 µm) and cytochalasin D (0.19 µm) but differed significantly after nocodazole (0.18 µm) and taxol (0.20 µm) washout (*p* < 0.05) (Figure 6C).

### 3.7. Cytoskeletal Dynamics Critically Determine the Proteolytic Activity of GM-BMDC Podosomes

**Figure 7:**
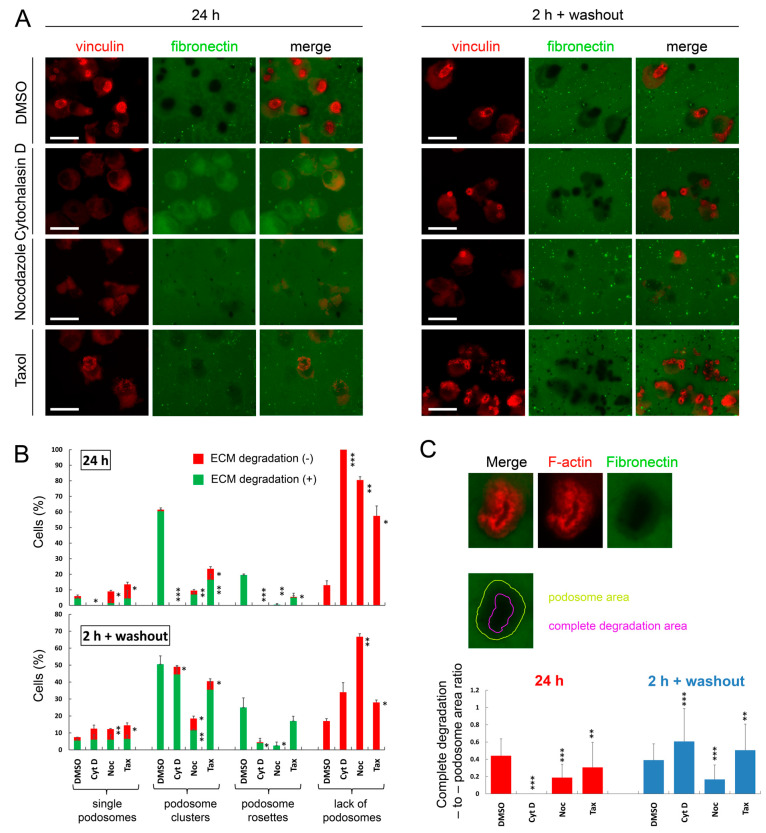
Cytoskeletal inhibitors-induced disassembly, reorganization, and partial disintegration of podosomes, with altered podosome-associated proteolytic activity in GM-BMDCs. (**A**) Fluorescence microscopy analysis of the colocalization of podosomes and the ECM protein fibronectin in cells. Cells treated with DMSO (control), cytochalasin D, nocodazole, or taxol were stained for vinculin (red) and fibronectin (green) after 24 h of treatment and after 2 h, followed by washout. Areas of fibronectin signal loss indicate sites of ECM degradation. Original magnification: 1500×. Scale bars: 20 µm. (**B**) Quantitative analysis of cells exhibiting podosome-dependent ECM degradation and classification of podosome organization. The mean percentage of cells lacking podosomes or containing single podosomes, clusters, or rosettes in control and inhibitor-treated cells is shown after 24 h and after 2 h followed by washout. (**C**) Quantitative analysis of the ratio of complete ECM degradation to podosome area in control and cytoskeletal inhibitor–treated cells after 24 h and after 2 h, followed by washout. The number of degradation areas was counted in at least 30 randomly selected cells per experimental condition from three independent experiments. Data represent mean values from three independent experiments. Statistical significance was determined using a paired or unpaired Student’s *t*-test. Asterisks next to the columns (**B**) or above the columns (**C**) indicate significant differences compared with the DMSO control at the corresponding time point: * *p* < 0.05, ** *p* < 0.01, *** *p* < 0.001.

To assess the functional relevance of the observed adhesion structures, we next analyzed extracellular matrix degradation capacity (Figure 7), focusing on GM-CSF-derived BMDCs, as our data demonstrated that they form significantly larger and more numerous podosomes than Flt3L-derived BMDCs.

After 24 h of culture, DMSO-treated GM-BMDCs exhibited a high proportion of podosome-bearing cells (>86%), predominantly clustered (>61%) and rosette-shaped (19%) structures, all displaying strong proteolytic activity and complete ECM degradation localized at podosome sites (Figure 7A,B). In contrast, cytochalasin D treatment abolished actin-based structures, including podosomes (*p* < 0.001), resulting in complete loss of ECM degradation (Figure 7A,B). Nocodazole treatment resulted in a predominance of podosome-negative cells (80%; *p* < 0.01), with only a minor fraction forming clustered podosomes (7%; *p* < 0.01) or single podosomes (7%; *p* < 0.05), most of which lacked proteolytic activity. Taxol treatment preserved the highest proportion of podosome-positive cells among inhibitors, with 43% bearing podosomes, including clustered (16.5%; *p* < 0.01), rosette (4%; *p* < 0.05), and single (9%; *p* < 0.05) forms, although a substantial fraction lacked ECM-degrading capacity (Figure 7A–C).

Following inhibitor washout after short-term exposure (2 h + 24 h culture), partial restoration of podosome formation and function was observed across all conditions (Figure 7A,B). In cytochalasin D-treated cells, podosomes reassembled in 65% of cells, predominantly as clustered structures (44%) with restored ECM degradation, while rosettes (4%) also reappeared with functional activity (*p* < 0.05). Nocodazole washout resulted in limited recovery, with >66% podosome-negative cells (*p* < 0.001), and only 11% of clustered podosomes retaining proteolytic function (*p* < 0.01). In contrast, taxol washout led to the most efficient recovery, with 72% of cells bearing podosomes, including clustered (40%) and rosette (17%) forms exhibiting strong ECM degradation, while only 5% of clustered structures lacked activity (Figure 7B). Single podosomes were observed in 10% of cells across conditions, with approximately half showing partial ECM degradation.

Quantitative analysis of ECM degradation efficiency, expressed as the ratio of degraded fibronectin area to podosome area, further confirmed these trends (Figure 7C). In control cells, the ratio reached 0.44. Continuous 24 h inhibitor treatment significantly reduced this parameter, particularly under nocodazole (0.18) and taxol (0.30), indicating impaired proteolytic function. Following washout, this ratio increased under cytochalasin D (0.60; *p* < 0.001) and taxol (0.50; *p* < 0.01), indicating partial functional recovery, whereas nocodazole-treated cells showed persistently reduced activity (0.16; *p* < 0.001 compared to control). Overall, short-term inhibitor exposure followed by washout promoted partial restoration of podosome-mediated ECM degradation, whereas continuous exposure led to sustained functional impairment, highlighting the dynamic dependence of podosome-mediated matrix degradation on cytoskeletal integrity.

### 3.8. Cytoskeletal Dynamics Critically Regulate GM-BMDC Migration in a Wound-Healing Assay

**Figure 8:**
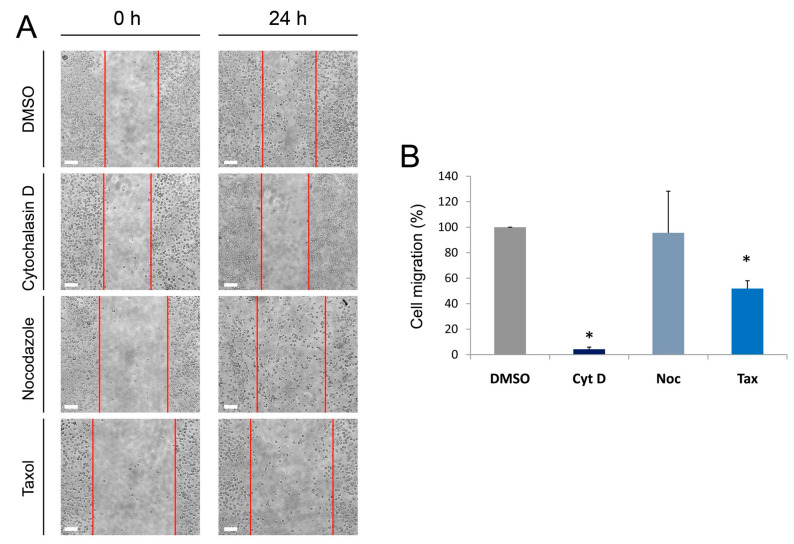
Cytoskeletal perturbation differentially affects GM-BMDC migration in a wound-healing assay. (**A**) Representative phase-contrast micrographs showing wound closure in a confluent cell monolayer at 0 h and 24 h after treatment with DMSO (control), cytochalasin D, nocodazole, or taxol. Red lines indicate the initial wound boundaries. Original magnification: 100×. Scale bars: 100 µm. (**B**) Graphs show the mean number of cells migrating into the wound area after 24 h ± standard deviation (SD) from three independent experiments. Cell migration was quantified as the difference between the number of cells that had entered the wound area at 24 h and the number of cells present in the wound area at 0 h. Statistical significance was determined using a paired Student’s *t*-test. Asterisks above the columns indicate significant differences compared with the DMSO control: * *p* < 0.05.

Because podosomes and FAs are key adhesive structures involved in DC migration; we assessed the migratory capacity of GM-BMDCs under cytoskeleton-interfering drugs using a wound-healing assay by quantifying cells entering the wound area after 24 h (Figure 8). Under control conditions, GM-BMDCs efficiently migrated and almost completely closed the wound within 24 h. In contrast, cytochalasin D strongly impaired migration, with fewer than five cells entering the wound area (*p* < 0.05), resulting in minimal wound closure. Interestingly, nocodazole treatment did not significantly affect migration, with 100 cells entering the wound area, comparable to DMSO controls (Figure 8A,B). Meanwhile, taxol significantly reduced migration but did not abolish it, with approximately 50 migrating cells observed (*p* < 0.05), leading to partial wound closure. Collectively, these results indicate that actin polymerization is essential for BMDC migration, whereas microtubule dynamics modulate migratory efficiency, with microtubule stabilization exerting a stronger inhibitory effect than depolymerization in 2D conditions. The lack of a significant effect of nocodazole suggests that microtubule depolymerization alone is not sufficient to impair GM-BMDC migration in a 2D wound-healing context, likely due to compensatory actin-driven motility mechanisms.

## 4. Discussion

DCs are highly dynamic immune cells whose migratory capacity and interaction with the microenvironment are essential for their function as APCs [18,19]. Cell migration is a fundamental biological process required for embryogenesis, immune surveillance, and tissue homeostasis. Its dysregulation contributes to numerous pathologies, including cancer progression and metastasis. Migration is a multistep and tightly coordinated process initiated by membrane protrusion, which is driven by spatially and temporally regulated actin polymerization at the leading edge [20,21]. This process is critically dependent on the cytoskeleton, which integrates actin dynamics with microtubule-mediated regulation of polarity, adhesion turnover, and intracellular trafficking, thereby coordinating both protrusive activity and cell–substrate interactions during migration [20].

Migratory protrusions, including lamellipodia, filopodia, and podosome/invadopodia-like structures, differ in morphology and function but all rely on coordinated cytoskeletal remodeling [20,21]. In DCs, this cytoskeletal plasticity is particularly important due to their need to rapidly adapt migratory behavior in response to environmental cues and to traverse complex tissue environments, including endothelial and lymphatic barriers [22]. Thus, both adhesion structures and the cytoskeleton are key determinants of DC motility and functional competence. Therefore, understanding the molecular basis of DCs migration is critical for elucidating both their physiological roles and mechanisms underlying DC-related pathologies.

Importantly, GM-CSF- and Flt3L-derived bone marrow cells are widely used in vitro models of DCs; however, the organization of their adhesion structures is not well characterized. It is unclear how podosomes and focal adhesions are spatially organized in these two cell models and how they are regulated by cytoskeletal components. Therefore, in the present study we performed a systematic comparative analysis of GM-BMDCs and FL-BMDCs to characterize their adhesion structure architecture and organization.

Our findings demonstrate that actin filaments and microtubules serve functionally distinct yet complementary roles in the regulation of DC adhesion structures. While the actin cytoskeleton provides the structural foundation for podosome and FA formation and maintenance, microtubules act as higher-order regulators of their spatial organization and dynamics. Microtubules are key regulators of cell adhesion and migration, primarily through their roles in adhesion turnover and intracellular signaling [23]. Their transport and signaling functions, in concert with interactions with other cytoskeletal components, establish a complex regulatory network controlling the dynamics and function of both podosomes and FAs [24].

Analysis of microtubule organization in control (DMSO) GM-BMDCs and FL-BMDCs revealed a radially organized network emanating from the MTOC. Such architecture promotes cell polarization and efficient migration by enabling directional transport of vesicles, signaling proteins, and cytoskeletal components to the leading edge [24,25,26,27]. Disruption of microtubule dynamics, via depolymerization (nocodazole) or stabilization (taxol), led to significant reorganization of the microtubule network and loss of its characteristic MTOC-dependent architecture in both cell models. Importantly, these changes were reversible upon inhibitor removal, indicating high cytoskeletal plasticity. These findings underscore that the dynamic balance between microtubule polymerization and depolymerization is essential for maintaining cellular spatial organization and integrating migration-regulating signals, including adhesion-dependent processes.

The cytoskeleton forms a complex, dynamic network of protein filaments that provides structural support, maintains cell shape, and participates in intracellular transport, migration, and division [28]. It also regulates cell–ECM signaling, controlling the organization, dynamics, function, and force-generating capacity of adhesion structures such as podosomes and FAs [29,30]. Podosomes are specialized actin- and integrin-dependent adhesion structures that mediate cell adhesion and ECM degradation. Their formation is initiated by branched actin network assembly stabilized by cortactin, facilitating lamellipodial protrusion, the primary source of migratory forces. Podosomes localize predominantly within lamellipodia, initiated via the N-WASP (neural Wiskott–Aldrich syndrome protein)/WASP (Wiskott–Aldrich syndrome protein) complex activating Arp2/3 (actin-related protein 2/3) and cortactin through integrin-dependent signaling. Maturation involves recruitment of podosome components and actin network remodeling via cofilin. Mature podosomes concentrate proteolytic enzymes for ECM degradation, and continuous activity of N-WASP/Arp2/3, cortactin, and cofilin maintains podosome integrity through ongoing actin polymerization [20,31,32].

In control (DMSO) GM-BMDCs and FL-BMDCs, podosomes exhibited classical organization with an actin core surrounded by adaptor proteins such as vinculin. With increasing adhesion time, these structures progressively reorganized from single podosomes to podosome clusters and rosettes. More complex structures correlate with higher proteolytic activity and ECM remodeling efficiency, as previously observed in endothelial cells [33] and fibroblasts [34]. Notably, GM-BMDCs displayed larger podosomes and a higher proportion of cells containing these structures, which may be associated with differences in cytoskeleton-dependent adhesive and migratory functions.

Direct depolymerization of actin filaments using cytochalasin D nearly abolished podosomes in both cell models, demonstrating the fundamental role of actin in podosome formation and maintenance [15,35]. This mechanism results from blocking G-actin incorporation, inhibiting polymerization and destabilizing actin filaments [36]. Similar effects were observed with latrunculin B in endothelial cells [37], reinforcing that continuous actin turnover is necessary for structural integrity. In contrast, microtubule perturbations affected podosomes indirectly. They did not eliminate podosomes but disrupted their organization and reduced the formation of complex structures such as rosettes. These results indicate that microtubules primarily regulate podosome dynamics and organization through transport of components and modulation of local actin dynamics. Literature supports this interpretation: microtubule plus-ends and KIF1C (kinesin family member 1C) directly regulate podosome dynamics in macrophages [38], and similar effects occur in osteoclasts, where microtubule disruption impairs podosome belt formation [39]. Microtubule instability modulates Rho GTPase signaling, influencing podosome localization and dynamics [29,40].

A key aspect of this study is the direct comparison of GM-BMDCs and FL-BMDCs. Our data indicate that adhesion structure organization correlates closely with cellular functional phenotype. GM-BMDCs exhibited larger, more numerous podosomes with high proteolytic activity, consistent with an inflammatory-like phenotype. In contrast, FL-BMDCs, more reflective of physiological in vivo DC populations, contained fewer podosomes and a higher proportion of stable FAs, suggesting alternative migratory strategies and ECM interactions.

Proper coordination between actin filaments and microtubules proved critical not only for podosome organization but also for proteolytic functionality. Colocalization of podosomes in GM-BMDCs with ECM degradation zones confirmed their direct involvement in microenvironment remodeling, consistent with concentrated metalloproteinase activity observed in macrophage podosomes [41]. Cytoskeletal disruption significantly impaired this function, particularly actin depolymerization, which completely abolished proteolytic activity. Microtubule inhibitors also reduced ECM degradation, although effects were less pronounced and partially reversible, highlighting a regulatory rather than structural role [29]. These findings are in line with previous work [42] demonstrating that podosomes are critical for the mesenchymal-like migration of human GM-CSF-derived DCs in 3D environments. In that study, the ability to form podosomes was shown to be independent of the maturation status of DCs, while podosome-mediated matrix degradation facilitated migration through dense extracellular matrices, highlighting the importance of these structures for invasive migration [42].

FAs represent a second major actin-dependent adhesion structure. In both analyzed DC subsets, they were small, peripheral, and colocalized with actin stress fibers, stabilizing adhesion and regulating directional migration through assembly at the leading edge and disassembly at the rear [27,43]. Actin filaments initiate FA formation via lamellipodia protrusion and integrin activation. Stress fiber formation, RhoA-dependent, generates actomyosin tension that promotes FAs maturation and adaptor recruitment (talin, vinculin). Mechanical forces transmitted through actin filaments, coupled with actin network remodeling, stabilize FAs size and turnover, essential for effective migration [1,30,44]. Our results support this model: actin integrity was essential for maintaining FA organization, as actin depolymerization nearly completely abolished FA formation. Microtubules regulate FA dynamics through adaptor proteins such as KANK1 (KN motif and ankyrin repeat domain-containing protein 1) and +TIP (plus-end tracking proteins) family members, which stabilize microtubule plus-ends. In addition, the release of GEF-H1 (guanine nucleotide exchange factor H1) from depolymerized microtubules activates RhoA, thereby increasing actomyosin contractility and promoting FA formation [45].

Microtubules also serve as tracks for the intracellular transport of integrins, exocyst components, and metalloproteinases (e.g., MT1-MMP), facilitating both FA assembly and disassembly [27,46]. Disruption of microtubule dynamics did not abolish FAs, but significantly altered their size and morphology. Both depolymerization and excessive stabilization led to increased FA size and stability, likely due to impaired microtubule-dependent transport required for adhesion turnover. Together, these findings highlight that FA dynamics rely on coordinated interactions between actin and microtubules: actin provides structural support, whereas microtubules regulate turnover and spatial organization [47,48]. Consistent with our observations, nocodazole-induced microtubule depolymerization in HeLa cells was shown to enhance RhoA-dependent contractility and promote FA reorganization, underscoring the link between microtubule dynamics and cytoskeletal regulation [45]. In NIH3T3 fibroblasts, nocodazole-induced microtubule depolymerization was likewise associated with increased FA stability and number, whereas restoration of the microtubule network following nocodazole washout promoted rapid FA disassembly, indicating a critical role of microtubules in FA turnover [47]. Moreover, disruption of the microtubule network impairs intracellular transport of endocytic components, such as clathrin, which are essential for FA disassembly, thereby further limiting adhesion turnover [47,48]. In an ovarian cancer cell model, taxol treatment was shown to inhibit FAK activity, which was associated with the presence of larger and more stable focal adhesions and reduced FA remodeling. These changes correlated with altered microtubule dynamics and decreased migratory capacity [49].

Migration of DCs is critical for immune responses, enabling antigen transport to lymph nodes and subsequent T-cell activation [50,51,52]. Our data demonstrate that cytoskeletal dynamics are essential for this process. Actin depolymerization completely abolished GM-BMDC migration, confirming its key role in generating migratory forces. In contrast, microtubule stabilization with taxol partially impaired migration, indicating that increased cytoskeletal rigidity limits the dynamic remodeling required for efficient movement. Microtubule depolymerization had a minimal effect on 2D migration, suggesting that actin-driven motility is dominant in this context. These findings are consistent with previous reports showing that microtubule perturbation can differentially affect cell motility. For example, in wild-type CHO and HeLa cells, microtubule depolymerization does not abolish migration but disrupts polarity and directionality, highlighting their role in spatial coordination rather than force generation [53]. Similarly, Liao et al. (1995) demonstrated that even low concentrations of nocodazole, which minimally affect microtubule mass, significantly reduce migration speed. Sub-polymerizing doses of taxol and vinblastine also impair fibroblast motility, indicating that dynamic microtubule remodeling is critical for efficient migration [54]. In tumor and endothelial models, both microtubule- and actin-targeting agents significantly reduce migration and wound closure capacity [55,56]. The reduced migration of GM-BMDCs upon microtubule inhibition likely reflects impaired focal adhesion dynamics, including altered adhesion turnover and stability, which disrupts the coordinated cycles of attachment and detachment required for efficient cell movement.

In summary, our findings highlight the critical importance of proper cytoskeletal regulation for coordinating adhesion structure organization and function in DCs. Actin filaments provide structural support for podosome and FAs formation and maintenance, while microtubules regulate their dynamics, spatial organization, and component transport. Imbalances between stability and dynamics of these elements lead to dysregulated adhesion structures, directly impairing migratory and proteolytic capacities. Observed differences between GM-BMDCs and FL-BMDCs further emphasize the significance of cellular context in DC migration studies and the importance of model choice for interpreting the experimental results.

## 5. Conclusions

This study demonstrates that cytoskeletal organization is a key determinant of DC adhesion structure formation, ECM degradation, and migratory behavior. GM-CSF- and Flt3L-derived BMDCs represent distinct in vitro DC models that differ in the abundance and organization of podosomes and focal adhesions, reflecting differences in cytoskeletal architecture and functional plasticity. Among them, GM-BMDCs display a more robust and consistent podosome-forming phenotype, making them better suited for quantitative analyses of adhesion-dependent functions.

The limitations of this study include the use of 2D in vitro models, which do not fully recapitulate the in vivo tissue environment. DC migration in 3D may employ additional mechanisms independent of classical adhesion structures. Moreover, BMDC models, while widely used, do not capture the full heterogeneity of in vivo DC populations. Future studies should therefore extend these analyses to 3D matrices and in vivo models to better define how adhesion structures function in physiologically relevant environments. Investigating DC migration within confined tissues and lymphatic barriers may reveal additional cytoskeleton-independent or alternative adhesion mechanisms. Furthermore, comparative studies across primary DC subsets could help to better resolve the functional diversity of adhesion systems observed in vitro.

## Data Availability

All data generated or analyzed during this study are included in this published article.

## Abbreviations

The following abbreviations are used in this manuscript:

APC	Antigen presenting cell
BMDCs	Bone marrow-derived DCs
cDC	Conventional DC
DC	Dendritic dell
FA	Focal adhesion
FL-BMDCs	Flt3L-derived BMDCs
GM-BMDCs	GM-CSF-derived BMDCs
GM-CSF	Granulocyte-macrophage colony-stimulating factor
MTOC	Microtubule organizing center
